# Autophagy-Related Gene *PlATG6a* Is Involved in Mycelial Growth, Asexual Reproduction and Tolerance to Salt and Oxidative Stresses in *Peronophythora* *litchii*

**DOI:** 10.3390/ijms23031839

**Published:** 2022-02-06

**Authors:** Jingrui Wang, Gangqiang Zhou, Weixiong Huang, Wen Li, Dinan Feng, Liuchen Liu, Pinggen Xi, Zide Jiang, Guanghui Kong

**Affiliations:** Department of Plant Pathology/Guangdong Province Key Laboratory of Microbial Signals and Disease Control, South China Agricultural University, Guangzhou 510642, China; wangjr202103@163.com (J.W.); 20212021023@stu.scau.edu.cn (G.Z.); qq184863352@163.com (W.H.); wenli@stu.scau.edu.cn (W.L.); fengdn08@163.com (D.F.); liu_liuchen@stu.scau.edu.cn (L.L.); xpg@scau.edu.cn (P.X.); zdjiang@scau.edu.cn (Z.J.)

**Keywords:** autophagy-related gene, ATG6, *Peronophythora litchii*, sporangium production, mycelial growth, pathogenicity, oxidative stress

## Abstract

Autophagy is ubiquitously present in eukaryotes. During this process, intracellular proteins and some waste organelles are transported into lysosomes or vacuoles for degradation, which can be reused by the cell to guarantee normal cellular metabolism. However, the function of autophagy-related (ATG) proteins in oomycetes is rarely known. In this study, we identified an autophagy-related gene, *PlATG6a,* encoding a 514-amino-acid protein in *Peronophythora litchii,* which is the most destructive pathogen of litchi. The transcriptional level of *PlATG6a* was relatively higher in mycelium, sporangia, zoospores and cysts. We generated *PlATG6a* knockout mutants using CRISPR/Cas9 technology. The *P. litchii* Δ*platg6a* mutants were significantly impaired in autophagy and vegetative growth. We further found that the Δ*platg6a* mutants displayed decreased branches of sporangiophore, leading to impaired sporangium production. PlATG6a is also involved in resistance to oxidative and salt stresses, but not in sexual reproduction. The transcription of peroxidase-encoding genes was down-regulated in Δ*platg6a* mutants, which is likely responsible for hypersensitivity to oxidative stress. Compared with the wild-type strain, the Δ*platg6a* mutants showed reduced virulence when inoculated on the litchi leaves using mycelia plugs. Overall, these results suggest a critical role for PlATG6a in autophagy, vegetative growth, sporangium production, sporangiophore development, zoospore release, pathogenesis and tolerance to salt and oxidative stresses in *P. litchii*.

## 1. Introduction

Oomycetes are a class of ubiquitous filamentous eukaryotic microorganisms, which are evolutionarily close to photosynthetic algae [1]. Many oomycetes are economically significant pathogens affecting agriculture, forestry and the ecosystem. For example, *Phytophthora infestans*, *P. sojae*, *P. capsici* and *Peronophythora litchii* severely damage potato, soybean, cucurbits, and litchi, respectively [2,3]. Among them, litchi downy blight caused by *P. litchii* is the most destructive disease of litchi, leading to 30% to 80% yield losses annually [3]. However, the molecular mechanisms of *P. litchii* growth, development and pathogenesis are largely unknown [4].

Autophagy is a conserved cellular process and is essential for cell survival under various stressful conditions (e.g., starvation), in which cytoplasmic contents are degraded within a lysosome or vacuole, and the resulting micromolecular constituents are recycled [5,6,7]. Recent studies have revealed a wide variety of physiological roles for autophagy involved in pathogens and plants [8]. In many eukaryotic plant pathogens, such as *Magnaporthe oryzae*, *P. sojae*, *Ustilago maydis* and *Fusarium graminearum*, autophagy is associated with sporulation, virulence and development [9,10,11,12,13,14,15,16].

As a key regulator of autophagy, Atg6/Vps30 (VPS, vacuolar protein sorting) is a subunit of different phosphatidylinositol 3-kinase (PI3K) complexes involved in either autophagy or the vacuolar protein sorting pathway [17]. In yeast, Atg6 can form two different PI3K complexes, type I and type II, which function in autophagy and vacuolar protein sorting, respectively [18]. In *Cryptococcus neoformans*, Atg6 was found to be involved in thermotolerance, oxidative stress tolerance and laccase activity [19]. In *M. oryzae*, deletion of *MoATG6* severely impaired sporulation and pathogenicity [20]. MoAtg6 is recruited by Vps34 to the pre-autophagosomal structure (PAS) and thus promotes autophagy activity in *M. oryzae* [21]. In oomycetes, the autophagy process is required for *P. infestans* during asexual development [22]. Twenty-six autophagy-related (ATG) genes were identified in *P. sojae*. It has been shown that silencing of *PsATG6a* impaired sporangium (or zoosporangium) production and pathogenesis [15]. However, the mechanisms of the ATG genes involved in the growth, development and pathogenesis of oomycetes are largely unclear.

In this study, we identified and functionally characterized a *P. litchii* ATG gene, *PlATG6a*, which is up-regulated in zoospores and cysts. We deleted *PlATG6a* and found the impairment of autophagy in Δ*platg6a* mutants. To investigate the potential function of autophagy in *P. litchii*, we evaluated the phenotype and found that Δ*platg6a* mutants showed defects in vegetative growth, sporangium production and tolerance to salt stress. Furthermore, we found that the sporangiophore of the Δ*platg6a* mutant produced fewer branches, leading to decreased sporangium production. The virulence of Δ*platg6a* mutants was reduced when inoculated on litchi leaves using mycelium plugs. We also found that *PlATG6a* could be induced by H_2_O_2_. Correspondingly, the Δ*platg6a* mutants showed impaired resistance to H_2_O_2_, and the transcriptional level of peroxidase-encoding genes were relatively lower in the Δ*platg6a* mutants. This study provided new insight into the functions of PlATG6a in autophagy, growth, development, pathogenesis and tolerance to salt and oxidative stresses in *P. litchii*.

## 2. Results

### 2.1. PlATG6a and Its Orthologs Are Widespread in Oomycetes, and PlATG6 Is Up-Regulated in Zoospores and Cysts in Peronophythora litchii

**Figure 1:**
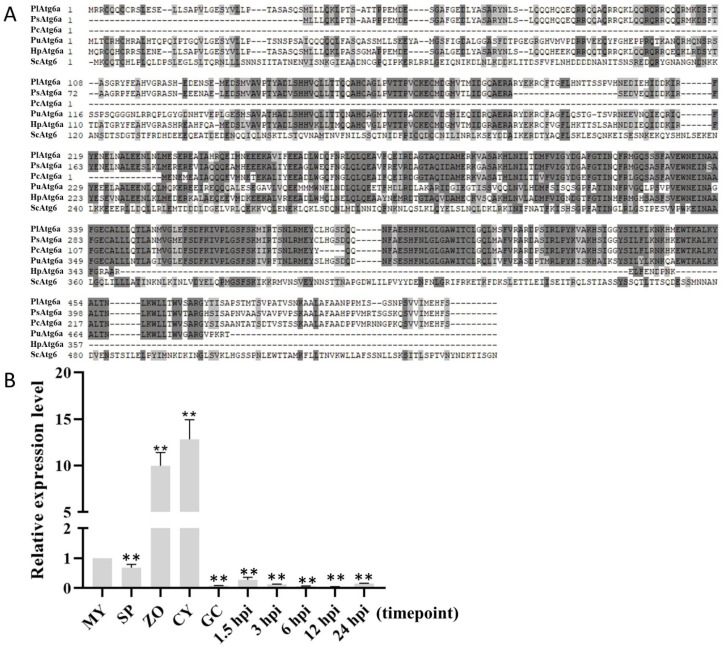
PlATG6a is conserved in oomycetes and up-regulated in the asexual stage of *Peronophythora litchii*. (**A**) Amino acid sequence alignment of PlATG6a and its orthologs from *Phytophthora sojae* (Ps), *P. capsici* (Pc), *Hyaloperonospora parasitica* (Hp), *Pythium ultimum* (Pu), and *Saccharomyces cerevisiae* (Sc). (**B**) Expression pattern of *PlATG6a* during the asexual life cycle and infection stages was analyzed by quantitative reverse transcription PCR (qRT-PCR). MY: mycelia; SP: sporangia; ZO: zoospore; CY: cyst; GC: germination of cyst; hpi: hours post inoculation. Relative expression levels were calculated using the 2^−ΔΔCT^ method [23] with *PlActin* gene as the internal control. The MY value was set as “1”. Asterisks indicate significant difference compared with MY (** *p* < 0.01, *t*-test). These experiments were repeated three times.

By BLAST searching using PsAtg6a sequence as a bait, we identified an Atg6a ortholog in *P. litchii*, hereinafter named PlAtg6. We searched the orthologs of *PlATG6a* in oomycete species, including *P. sojae*, *P. capsici, Pythium ultimum* and *Hyaloperonospora* *parasitica*. Orthologs of *PlATG6a* could be found in all these species (Supplementary Table S2), indicating that *PlATG6a* orthologs are widespread in oomycetes (Figure 1A).

Transcriptional analysis showed that the *PlATG6a* transcripts were significantly up-regulated in zoospores (ZOs) and cysts (CYs), and down-regulated during infection compared with mycelium (MY) (Figure 1B). The expression profile of *PlATG6a* was dramatically different from that of *PsATG6a,* which was up-regulated during infection. These results suggest that PlATG6a might function in the growth and asexual development stages. In this study, we focused on the function of PlATG6a.

### 2.2. Deletion of PlATG6a Affected Autophagy in P. litchii

**Figure 2:**
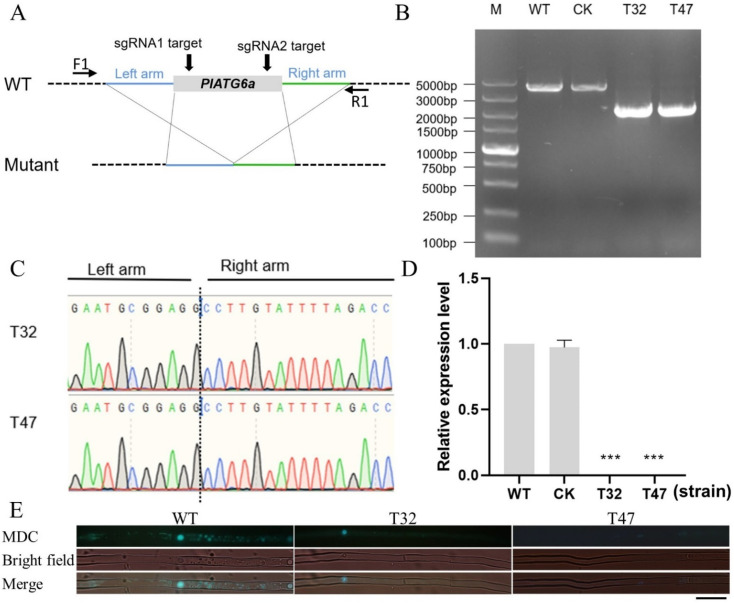
CRISPR/Cas9-mediated deletion of *PlATG6a* in *Peronophythora litchii*. (**A**) Schematic representation of the strategy of CRISPR/Cas9-mediated mutagenesis of *PlATG6a*. Two single-guide RNAs targeted the *PlATG6a* gene sequence (indicated by black arrows). The Δ*platg6a* mutants were identified by genomic PCR (**B**) and sequencing (**C**) with primers F1 and R1 (Supplementary Table S1). (**D**) Transcription of *PlATG6a* was analyzed by qRT-PCR in wild-type (WT) WT, CK and Δ*platg6a* mutants (T32 and T47). “***” indicates significant difference compared with WT (*p* < 0.001, *t*-test). These experiments were repeated three times. (**E**) Visualization of autophagosome by MDC staining. The WT and Δ*platg6a* mutants were incubated in carrot juice agar (CJA) medium for 3 days. After 3 washes and incubation with sterile distilled water for 4 h, hyphae samples were stained with MDC and analyzed by microscopy. Bars = 10 μm. WT: wild-type strain; CK: the transformant failed to acquire *PlATG6a* mutation (control).

To investigate the function of PlATG6a, we generated a deletion mutant of *PlATG6a* in *P. litchii* using CRISPR/Cas9 technology. Two single-guide RNAs were designed to disrupt the *PlATG6a* coding region (Figure 2A). The transformants were screened by G418 resistance and then verified by genomic PCR and sequencing (Figure 2B,C). Finally, two Δ*platg6a* mutants (T32, T47) were obtained, and a transformant that failed to delete the *PlATG6a* gene was selected as the control (CK). We also found that transcription of *PlATG6a* was undetected in T32 and T47, while it was detectable in the WT (wild-type strain, SHS3) and CK strains (Figure 2D), confirming successful deletion of this gene in T32 and T47 strains. In *P. litchii*, we observed more fluorescence in starved mycelia using monodansylcadaverine (MDC) staining (Figure S1), suggesting that autophagy occurred under starvation. Compared to WT, the Δ*platg6a* mutants showed a decreased accumulation of autophagosomes, as visualized by MDC staining, in hyphal cells (Figure 2E). Deletion of *PlATG6a* also resulted in a significant transcriptional change of more than half the ATG genes (Figure S2). These results suggest that the autophagy pathway was affected in the Δ*platg6a* mutants.

### 2.3. PlATG6a Is Required for Normal Hyphal Growth, Sporangium Production and Zoospore Release

**Figure 3:**
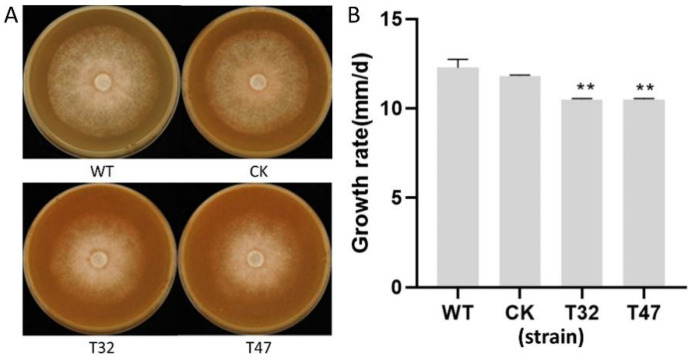
Growth rates of the wild-type (WT), CK (control; the transformant failed to acquire *PlATG6a* mutation), and the Δ*platg6a* mutants (T32 and T47). (**A**) The photographs were taken at 5 dpi. (**B**) The growth rate was measured and calculated. Bar chart depicts the mean ± SD. Asterisks represent significant difference compared with WT (** *p* < 0.01, n = 9, *t*-test).

To characterize the function of *PlATG6a* in *P. litchii* growth and differentiation, we first inoculated WT, CK and the Δ*platg6a* mutants on CJA medium and analyzed their colony morphology and hyphal growth at 5 days post inoculation (dpi). The Δ*platg6a* mutants showed significantly reduced growth rate on the CJA medium compared with that of WT (Figure 3A,B). This result indicates that PlATG6a is associated with vegetative growth in *P. litchii*.

**Figure 4:**
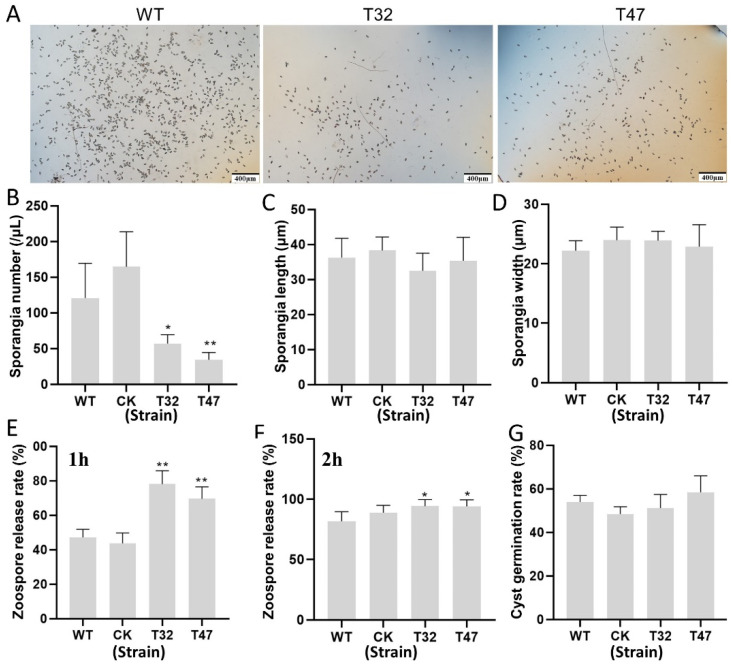
Knockout of *PlATG6a* impaired the sporangium production of *Peronophythora litchii* and promoted zoospore release. Δ*platg6a* mutants (T32 and T47) and WT were cultured on CJA medium for 5 days and sporangia were collected and used for releasing zoospores. (**A**) Photographs were taken after collecting sporangia from CJA medium. Scale bar = 400 μm. (**B**) The sporangia number was calculated. (**C**,**D**) The sporangia length and width were measured. (**E**,**F**) The zoospore release rates of Δ*platg6a* mutants, CK and WT were calculated at 1 and 2 h post incubation of the sporangia in water. (**G**) Cyst germination rate. The bar charts depict the means ± SDs. Asterisks indicate significant difference vs. WT (* *p* < 0.05, ** *p* < 0.01, *t*-test). These experiments were repeated three times.

In its asexual life cycle, *P. litchii* produces sporangia, which then release zoospores. Sporangia and zoospores play essential roles in both the initial stage of infection and the spread of oomycetes from host to host [4,24]. To investigate the function of *PlATG6a* in asexual development, we collected and calculated the number of sporangia of the Δ*platg6a* mutants, CK and WT grown on CJA medium at 25 °C for 5 days. We found that loss of *PlATG6a* significantly impaired the production of sporangia (Figure 4A,B), but did not affect sporangia morphology (Figure 4C,D). Zoospore release occurred when incubating the sporangia in water, and the rate was measured at 1 and 2 h post incubation. Our results showed that Δ*platg6a* mutants released more zoospores compared with WT, especially at 1 h after incubation (Figure 4E,F). Deletion of *PlATG6a* did not affect cyst germination rate (Figure 4G). These results indicate that *PlATG6a* affected the sporangium production and zoospore release.

### 2.4. PlATG6a Was Involved in the Morphology of Sporangiophore

**Figure 5:**
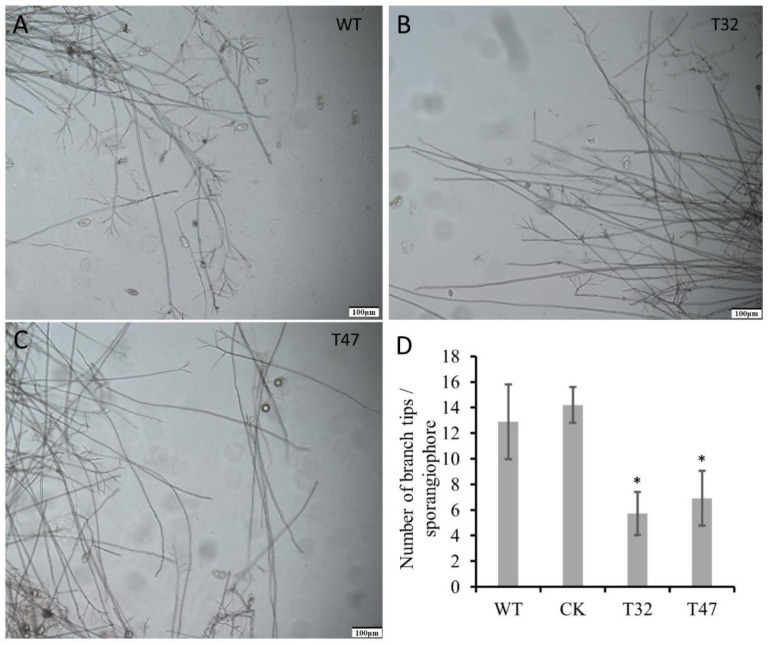
*PlATG6a* regulates sporangiophore morphology. (**A**–**C**) Microscopic images showing branches of sporangiophore. T32 and T47 are Δ*platg6a* mutants. (**D**) Number of branches per sporangiophore were calculated. Mean ± SD, derived from three independent biological repeats, for each strain. Asterisks represent a significant difference vs. WT (* *p* < 0.05) based on *t*-test.

To further investigate the mechanism of PlATG6a’s involvement in sporangium production, we next assessed the morphological characteristic of the Δ*platg6a* mutants. We found that each sporangiophore of the Δ*platg6a* mutants produced fewer branches, as compared with WT and CK strains (Figure 5). The result suggests that PlATG6a affects sporangium production, likely via contributing to the formation of sporangiophore branches.

### 2.5. Virulence of Δplatg6a Mutants

**Figure 6:**
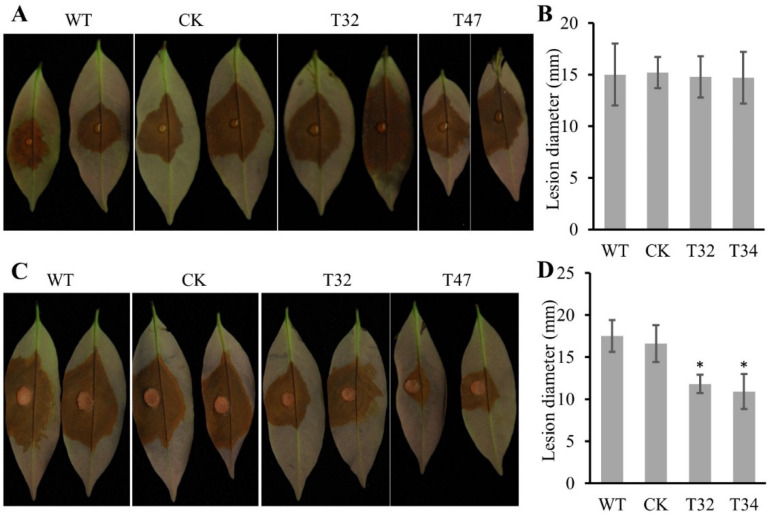
Virulence assessment. The virulence of Δ*platg6a* mutants was tested on litchi leaves, with WT and CK as controls. Zoospores (**A**) or mycelial plugs (**C**) were inoculated on the litchi leaves. Photographs were taken at 48 hpi. (**B**,**D**) The lesion length was measured at 48 hpi, corresponding to (**A**,**C**), respectively. CK: control; the transformant failed to acquire the *PIATG6a* mutation. Asterisks indicate significant difference vs. WT (* *p* < 0.05, *t*-test). These experiments were repeated three times, each containing 3 leaves for each strain.

We next tested the virulence of the Δ*platg6a* mutants, CK and WT using detached litchi leaves. There was no significant difference in virulence between the Δ*platg6a* mutants and WT or CK strains when litchi leaves were inoculated with zoospores of these strains (Figure 6A,B). In fungi, laccases participate in the oxidation of antibiotics such as flavonoids and phytoalexins, and thus contribute to the virulence of pathogens [25]. In oomycetes, laccase activity is also associated with plant infection [26,27,28]. Here we also tested the laccase activity of the Δ*platg6a* mutants T32 and T47 and found that they showed similar laccase activity as compared with WT and CK (Figure S3). Therefore, we conclude that loss of *PlATG6a* did not affect the virulence of *P. litchii* zoospores, which displayed normal laccase activity.

However, the Δ*platg6a* mutants produced fewer sporangia, which are reported to be important for initial infection [24]. Reduced virulence could be expected if we were to inoculate litchi leaves with mycelial plugs, because the Δ*platg6a* mycelial plugs carried fewer sporangia. Our results showed that the lesion length caused by the Δ*platg6a* mycelial plugs was reduced, compared with those caused by WT or CK mycelial plugs (Figure 6C,D). Therefore, PlATG6a is involved in plant infection, likely via regulating asexual development.

### 2.6. PlATG6a Is Involved in Tolerance to Salt and H_2_O_2_ Stress

**Figure 7:**
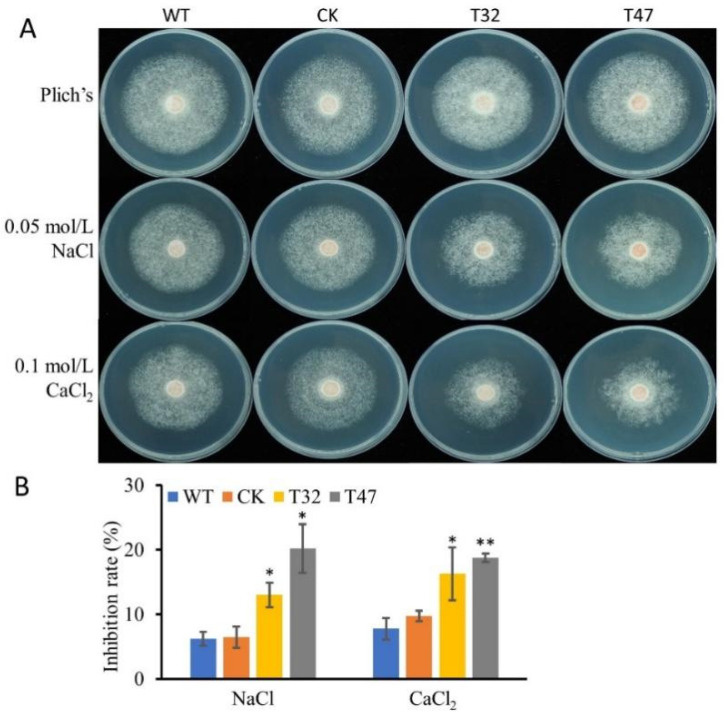
*PlATG6a* is involved in salt stress tolerance. (**A**) Mycelial of WT, CK and the Δ*platg6a* mutants (T32 and T47) grown on Plich medium with or without salt (0.05 M NaCl or 0.1 M CaCl_2_) supplement. Images were taken at 7 dpi. (**B**) Colony diameter was measured at 7 dpi. Growth inhibition rate (%) was calculated. Mean ± SD (n = 9 for each strain). Asterisks denote significant differences vs. WT (* *p* < 0.05; ** *p* < 0.01; *t*-test).

To investigate whether *PlATG6a* is related to tolerance to salt stress in *P. litchii*, the Δ*platg6a* mutants, WT and CK strains were grown on Plich medium supplemented with 0.05 M NaCl or 0.1 M CaCl_2_ for 7 days, before measurement of the colony diameter. The growth inhibition rates of the Δ*platg6a* mutants were significantly (*p* < 0.01) higher than those of WT or CK strains (Figure 7A,B), suggesting that *PlATG6a* may function in tolerance to salt stress.

**Figure 8:**
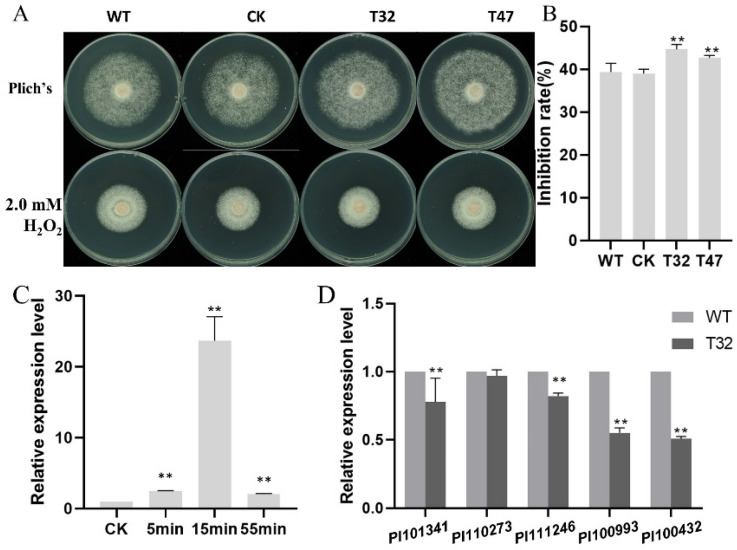
*PlATG6a* regulates response to oxidative stress in *Peronophythora litchii*. (**A**) WT, CK and the Δ*platg6a* mutants were allowed to grow on Plich medium with or without 2 mM H_2_O_2_ at 25 °C. Images were taken at 7 dpi. (**B**) The colony diameters were measured at 7 dpi. Growth inhibition rate (%) was calculated. WT and CK strains were used as controls. (**C**) Transcriptional analysis of the *PlATG6a* gene under oxidative stress (5 mM H_2_O_2_, for 0, 5, 15, 55 min). Expression levels were normalized using the values at 0 min as ‘1′. (**D**) qRT-PCR analysis of *P. litchii* putative peroxidase-encoding genes in Δ*platg6a* mutants and WT strain under oxidative stress conditions (5 mM H_2_O_2_, for 5 min). Data are mean ± SD (n = 9). Asterisks represent significant differences vs. WT (** *p* < 0.01) based on *t*-test.

In *C. neoformans*, the *atg6*Δ mutant was more sensitive to H_2_O_2_ treatment [19]. Peroxides are the signature products of the earliest defense responses of plants and play an important role in plant immunity against pathogens [29]. Here, we tested the sensitivity of the Δ*platg6a* mutants, using the CK and WT strains as the control. The results (Figure 8A,B) showed that the Δ*platg6a* mutants were significantly hypersensitive to the oxidative stress caused by H_2_O_2_ treatment, as compared to the WT or CK strains.

We next assessed the expression level of *PlATG6a* under oxidative stress, at different time points post exposure to H_2_O_2_, following the established method [15,30,31]. The result showed that transcription of *PlATG6a* was significantly up-regulated at 5 to 55 min after H_2_O_2_ treatment (Figure 8C), implying that this gene plays a role in response to oxidative stress. Furthermore, we examined the expression level of five selected peroxidase-encoding genes [31] in the WT strain and the Δ*platg6a* mutant. Four out of the five tested genes displayed a higher expression level in the WT strain than that in the mutant upon exposure to H_2_O_2_ for 5 min (Figure 8D). These results suggest that PlATG6a is involved in the oxidative stress response.

### 2.7. Knockout of PlATG6a Did Not Affect the Oospore Production of P. litchii

**Figure 9:**
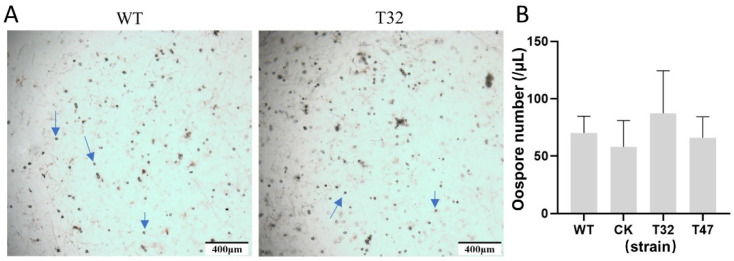
Assessment of sexual reproduction. (**A**) Photographs were taken at 10 dpi. The blue arrows indicate the representative oospores. (**B**) Oospore number was calculated. Data are mean ± SD (n = 9). There was no significant difference in oospore number between WT and the Δ*platg6a* mutant, T32 or T47 (*p* > 0.5; *t*-test).

To investigate the function of PlATG6a in the sexual stage, we examined the oospore production of WT and Δ*platg6a* mutants on CJA medium and found no significant difference between the Δ*platg6a* mutants and WT (Figure 9). In addition, we also found that the oospore size and morphological characteristics of the Δ*platg6a* mutant were not significantly different from WT. On the basis of these results, we infer that PlATG6a is not required for oospore formation. 

## 3. Discussion

As in other eukaryotes, autophagy-related (ATG) genes are conserved and play important roles in growth, development and pathogenicity in filamentous fungi [21]. Their specific roles need to be investigated in different organisms, considering the influence of evolutionary forces on autophagic processes [19]. In this study, we generated deletion mutants of the *PlATG6a* gene using CRISPR/Cas9 technology, for functional characterization. Our results suggest that PlATG6a is involved in autophagy and contributes to growth, asexual development and tolerance to oxidative stress in *P. litchii*.

*PlAGT6a* was up-regulated in zoospores and cysts, and therefore might be critical for asexual development, but not in the infection stage, which is different from the report that *PsATG6a* in *P. sojae* was up-regulated during the infection of a host plant [15]. We infer that this different transcriptional profile may reflect the different function of these two oomycete ATG6a proteins in growth and plant infection, although they share 84% similarity with each other [15].

The formation of the sporangiophore and its branches is very important for the asexual reproduction of many oomycetes, such as *P. litchii*, Peronosporaceae species and some *Phytophthora* species. The sporangiophore and branches determine the production of sporangia and zoospores, which is important for the spread of pathogens from host to host [24]. In this study, we found that knockout of *PlATG6a* impaired sporangium production. Our results are consistent with previous studies which found that ATG6a is involved in sporangium production in *P. sojae* and conidia formation in *M. oryzae* [15,20]. Here, we further revealed that the branches of the sporangiophore were reduced in the Δ*platg6a* mutants, likely accounting for impairment of sporangium production. However, the mechanism of ATG6a’s regulation of sporangium production may be not entirely the same in *P. litchii* and *P. sojae*, because there are hardly any sporangiophore branches produced by wild-type *P. sojae*. The mechanism of sporangiophore formation is not comprehensively known. A previous study reported that PlBZP32 negatively regulated the branch formation of sporangiophores [28]. Here, we reported that PlATG6 is a positive regulator in sporangiophore branch formation. Further study on the relationship between PlATG6a and PlBZP32 might provide a more detailed mechanism of asexual reproduction.

In *C. neoformans,* deletion of ATG6 affected laccase production, indicating that the Atg6-containing PI3K complex regulates laccase production [19]. It has also been shown that the *C. neoformans atg6*Δ mutant is sensitive to 10 mM H_2_O_2_ treatment [19]. It has not been reported whether the oomycete ATG genes play a role in regulating laccase activity or tolerance to oxidative stress. In this study, we reported for the first time that PlATG6 is involved in *P. litchii’s* response to oxidative stress. However, the deletion of *PlATG6a* did not affect laccase activity in *P. litchii*. These results indicate the functional differentiation of ATG6 orthologs in oomycetes and fungi.

*M. oryzae* ATG6 and *P. sojae* ATG6a are reported to be required for host plant infection [15,20]. Unlike PsATG6a, PlATG6a is not required for the virulence of *P. litchii* zoospores, although they share high similarity. Overall, our results display a functional differentiation of ATG6a orthologs in *P. sojae* and *P. litchii* during plant infection. When we inoculated litchi leaves with mycelial plugs, the lesion length caused by the Δ*platg6a* mutants was significantly reduced compared with WT and CK strains. We inferred that the reduced pathogenicity of the Δ*platg6a* mycelial plugs might be due to fewer sporangia being produced by the mutant. Meanwhile, the functional difference of PlATG6a and PsATG6a during lesion expansion should also be associated with the different transcriptional profiles of these two genes.

In *P. litchii*, PlATG6b (the ortholog of PsATG6b in *P. litchii*) shared 22% identity and 34% similarity with PlATG6a. Further study is required for the function of PlATG6b, and for the relationship between PlATG6a and PlATG6b.

This study adds new evidence that the autophagy process is required for the pathogenic development of oomycetous pathogens, besides the limited research on *P. infestans* and *P. sojae* that has previously been reported [15,22]. Further studies are needed to reveal how the autophagy process participates in the development and pathogenesis of oomycete pathogens.

In summary, we identified an ATG gene, *PlATG6a*, in *P. litchii* and demonstrated that PlATG6a is involved in growth, development, pathogenesis and tolerance to salt and oxidative stress. Furthermore, for the first time we reveal that PlATG6a is a positive regulator of sporangiophore branch formation. This study provides new insight into the mechanism of PlATG6a’s involvement in growth, development and oxidative stress in *P. litchii*.

## 4. Materials and Methods

### 4.1. Strains and Sequence

The *P. litchii* wild-type strain SHS3 (WT) was isolated and identified in this laboratory. *P. litchii* WT, CK and Δ*platg6a* mutants were maintained on carrot juice agar (CJA) medium (juice from 300 g carrot for 1 L medium, 15 g agar/L for solid media) at 25 °C in the dark. The control (CK) strain was a transformant that failed to knockout *PlATG6a*. The genome sequence and gene annotations of *P. litchii* were obtained from NCBI (BioProject ID: PRJNA290406) [32], other sequences were obtained from the JGI genome portal (https://genome.jgi.doe.gov/portal/ accessed on 24 January 2022) and previous study [15]. The amino acid sequence alignment was generated and adjusted in BioEdit (version 7.0.9.1).

### 4.2. Transcriptional Level Analysis

The total RNA of *P. litchii* was extracted using the All-In-One DNA/RNA Mini-preps Kit (Bio Basic, Shanghai, China). The FastKing RT Kit (TIANGEN, Beijing, China) was used for the first-strand cDNA synthesis. The transcriptional levels of *PlATG6a* were analyzed with quantitative reverse transcription PCR (qRT-PCR) using SYBR^®^ Premix Ex Taq^TM^ II (TaKaRa, Shiga, Japan). The *P. litchii* actin gene (*PlActin*) was used as a loading control [33], and the relative fold change was calculated using the 2^−ΔΔCT^ method [23]. The primers used for these analyses are listed in Supplementary Table S1.

### 4.3. Growth and Development Analysis

To test growth rate, *P. litchii* WT, CK and Δ*platg6a* mutants were inoculated on plates (diameter = 9 cm) containing 15 mL CJA medium and cultured at 25 °C in the dark. The colony diameter size was measured and the growth rate was calculated and photographed 5 days after inoculation. Statistical analysis was performed by *t*-test. The experiments were repeated three times.

For asexual development assays, the methods were described previously [31]. Five 9 mm diameter mycelial plugs were flushed with 2.5 mL double-sterilized water to obtain the sporangia suspension. Then the sporangia were purified using a 100 μm mesh filter. The sporangia were incubated at 16 °C for one hour for release of zoospores. Zoospores were encysted by shaking the suspension for 30 s on an oscillator. Cysts were incubated at 25 °C, 60 rpm for 1 or 2 h for germination. The number of oospores was measured as previously described [33].

### 4.4. CRISPR/Cas9 Gene Editing for PlATG6a

Two sgRNAs were designed and inserted into the sgRNA vector pYF2.3G-Ribo-sgRNA as previously described [34,35]. To generate gene-replacement constructs, 1 kb long upstream/downstream arms of the *PlATG6a* coding region were inserted into pBluescript II KS vector (Figure 3A). The pYF2.3G-RibosgRNA (*PlATG6a*) vector, the hSPCas9 vector pYF2-PsNLS-hSpCas9 and the pBluescript II KS (*PlATG6a*) vector were co-transformed into protoplasts of *P. litchii* by PEG-mediated protoplast transformation technology [34]. Preliminary transformants were screened by CJA medium containing 50 μg/mL G418. These transformants were further verified by genomic PCR and subsequent sequencing. These primers are listed in Supplementary Table S1.

### 4.5. Pathogenicity Assays

*P. litchii* WT, CK and mutants were inoculated on litchi leaves with 6 mm mycelial plugs or 100 zoospores. Then, the inoculated leaves were maintained at 25 °C in the dark. The diameter of the lesions was measured and calculated 48 h after inoculation. The significant differences were analyzed with *t*-tests. These experiments were repeated three times.

### 4.6. Sensitivity to Various Stress

To investigate the sensitivity of Δ*platg6a* mutants under different stress conditions, the mycelial plugs (diameter = 9 mm) of Δ*platg6a* mutants were inoculated in the center of the Plich medium [36]. The Plich media were supplemented with 0.05 mM NaCl or 0.1 mM CaCl_2_. The growth inhibition rate was calculated 7 days after inoculation at 25 °C in the dark. WT and CK strains were used as controls. Growth inhibition rate (%) = (growth diameter on stress-free plates—growth diameter on stress plates)/growth diameter on stress-free plates × 100%.

To analyze the expression levels of *PlATG6a* under oxidative stress, the WT strain was cultured in liquid Plich medium for 3 days at 25 °C in the dark. Then, the mycelia were immersed in the liquid medium supplemented with 5 mM H_2_O_2_ for 0, 5, 15 or 55 min. All samples were harvested and the expressional levels of *PlATG6a* were evaluated by qRT-PCR.

### 4.7. Microscopic Observation and Monodansylcadaverine (MDC) Staining

The microscopic observation was conducted with an Olympus BX53F microscope. MDC staining was performed as previously described [5].

## Data Availability

Not applicable.

## Supplementary Materials

The following supporting information can be downloaded at: https://www.mdpi.com/article/10.3390/ijms23031839/s1.

## References

[1] Cavalier-Smith T., Chao E.E., Lewis R. (2018). Multigene phylogeny and cell evolution of chromist infrakingdom Rhizaria: Contrasting cell organisation of sister phyla Cercozoa and Retaria. Protoplasma.

[2] Cázares-García S.V., Vázquez-Garcidueñas M.S., Vázquez-Marrufo G. (2013). Structural and Phylogenetic Analysis of Laccases from Trichoderma: A Bioinformatic Approach. PLoS ONE.

[3] Chen L., Zhang X., Wang W., Geng X., Shi Y., Na R., Dou D., Li H. (2017). Network and role analysis of autophagy in *Phytophthora sojae*. Sci. Rep..

[4] Chen X.R., Wang X.L., Zhang Z.G., Wang Y.C., Zheng X.B. (2008). Differences in the induction of the oxidative burst in compatible and incompatible interactions of soybean and *Phytophthora sojae*. Physiol. Mol. Plant Pathol..

[5] Contento A.L., Xiong Y., Bassham D.C. (2005). Visualization of autophagy in Arabidopsis using the fluorescent dye monodansylcadaverine and a GFP-AtATG8e fusion protein. Plant J..

[6] Deng Y.Z., Ramos-Pamplona M., Naqvi N.I. (2009). Autophagy-assisted glycogen catabolism regulates asexual differentiation in Magnaporthe oryzae. Autophagy.

[7] Fang Y., Tyler B.M. (2016). Efficient disruption and replacement of an effector gene in the oomycete *Phytophthora sojae* using CRISPR/Cas9. Mol. Plant Pathol..

[8] Feng Y., He D., Yao Z., Klionsky D.J. (2014). The machinery of macroautophagy. Cell Res..

[9] He C.C., Klionsky D.J. (2009). Regulation Mechanisms and Signaling Pathways of Autophagy. Annu. Rev. Genet..

[10] Huang J., Xi P., Deng Y., Huang W., Wang J., Zhao Q., Yang W., Li W., Situ J., Jiang L. (2021). The Mitogen-Activated Protein Kinase PlMAPK2 Is Involved in Zoosporogenesis and Pathogenicity of *Peronophythora litchii*. Int. J. Mol. Sci..

[11] Jiang L., Situ J., Deng Y.Z., Wan L., Xu D., Chen Y., Xi P., Jiang Z. (2018). PlMAPK10, a Mitogen-Activated Protein Kinase (MAPK) in *Peronophythora litchii*, Is Required for Mycelial Growth, Sporulation, Laccase Activity, and Plant Infection. Front. Microbiol..

[12] Jiang L., Ye W., Situ J., Chen Y., Yang X., Kong G., Liu Y., Tinashe R.J., Xi P., Wang Y. (2017). A Puf RNA-binding protein encoding gene *PlM90* regulates the sexual and asexual life stages of the litchi downy blight pathogen *Peronophythora litchii*. Fungal Genet. Biol..

[13] Judelson H.S., Blanco F.A. (2005). The spores of *Phytophthora*: Weapons of the plant destroyer. Nat. Rev. Microbiol..

[14] Kametaka S., Okano T., Ohsumi M., Ohsumi Y. (1998). Apg14p and Apg6/Vps30p form a protein complex essential for autophagy in the yeast, *Saccharomyces cerevisiae*. J. Biol. Chem..

[15] Kamoun S., Furzer O., Jones J.D.G., Judelson H.S., Ali G.S., Dalio R.J.D., Roy S.G., Schena L., Zambounis A., Panabières F. (2015). The Top 10 oomycete pathogens in molecular plant pathology. Mol. Plant Pathol..

[16] Kershaw M.J., Talbot N.J. (2009). Genome-wide functional analysis reveals that infection-associated fungal autophagy is necessary for rice blast disease. Proc. Natl. Acad. Sci. USA.

[17] Khan I.A., Lu J.P., Liu X.H., Rehman A., Lin F.C. (2012). Multifunction of autophagy-related genes in filamentous fungi. Microbiol. Res..

[18] Klionsky D.J., Baehrecke E.H., Brumell J.H., Chu C.T., Codogno P., Cuervo A.M., Debnath J., Deretic V., Elazar Z., Eskelinen E.-L. (2011). A comprehensive glossary of autophagy-related molecules and processes (2nd edition). Autophagy.

[19] Kong G., Chen Y., Deng Y., Feng D., Jiang L., Wan L., Li M., Jiang Z., Xi P. (2020). The Basic Leucine Zipper Transcription Factor PlBZP32 Associated with the Oxidative Stress Response Is Critical for Pathogenicity of the Lychee Downy Blight Oomycete *Peronophythora litchii*. mSphere.

[20] Kong G., Li T., Huang W., Li M., Shen W., Jiang L., Hsiang T., Jiang Z., Xi P. (2021). Detection of *Peronophythora litchii* on lychee by loop-mediated isothermal amplification assay. Crop. Prot..

[21] Li W., Li P., Zhou X., Situ J., Lin Y., Qiu J., Yuan Y., Xi P., Jiang Z., Kong G. (2021). A Cytochrome B5-Like Heme/Steroid Binding Domain Protein, PlCB5L1, Regulates Mycelial Growth, Pathogenicity and Oxidative Stress Tolerance in Peronophythora litchii. Front. Plant Sci..

[22] Liu X.H., Xu F., Snyder J.H., Shi H.B., Lu J.P., Lin F.C. (2016). Autophagy in plant pathogenic fungi. Semin Cell Dev. Biol..

[23] Livak K.J., Schmittgen T.D. (2001). Analysis of relative gene expression data using real-time quantitative PCR and the 2(-Delta Delta C(T)) Method. Methods.

[24] Luo Q., Wang F.X., Zhong N.Q., Wang H.Y., Xia G.X. (2014). The role of autophagy during development of the oomycete pathogen *Phytophthora infestans*. J. Genet. Genomics.

[25] Nadal M., Gold S.E. (2010). The autophagy genes atg8 and atg1 affect morphogenesis and pathogenicity in *Ustilago maydis*. Mol. Plant Pathol..

[26] Nguyen L.N., Bormann J., Le G.T., Stärkel C., Olsson S., Nosanchuk J.D., Giese H., Schäfer W. (2011). Autophagy-related lipase FgATG15 of *Fusarium graminearum* is important for lipid turnover and plant infection. Fungal Genet. Biol..

[27] Ruck A., Attonito J., Garces K.T., Núnez L., Palmisano N.J., Rubel Z., Bai Z., Nguyen K.C., Sun L., Grant B.D. (2011). The Atg6/Vps30/Beclin 1 ortholog BEC-1 mediates endocytic retrograde transport in addition to autophagy in *C. elegans*. Autophagy.

[28] Sheng Y., Wang Y., Meijer H.J., Yang X., Hua C., Ye W., Tao K., Liu X., Govers F., Wang Y. (2015). The heat shock transcription factor PsHSF1 of *Phytophthora sojae* is required for oxidative stress tolerance and detoxifying the plant oxidative burst. Environ. Microbiol..

[29] Situ J., Jiang L., Fan X., Yang W., Li W., Xi P., Deng Y., Kong G., Jiang Z. (2020). An RXLR effector PlAvh142 from *Peronophythora litchii* triggers plant cell death and contributes to virulence. Mol. Plant Pathol..

[30] Talbot N.J., Kershaw M.J. (2009). The emerging role of autophagy in plant pathogen attack and host defence. Curr. Opin. Plant Biol..

[31] van West P.V., Kamoun S., van’t Klooster J.W., Govers F. (1999). Internuclear gene silencing in *Phytophthora infestans*. Mol. Cell.

[32] Veneault-Fourrey C., Barooah M., Egan M., Wakley G., Talbot N.J. (2006). Autophagic fungal cell death is necessary for infection by the rice blast fungus. Science.

[33] Ye W., Wang Y., Shen D., Li D., Pu T., Jiang Z., Zhang Z., Zheng X., Tyler B.M., Wang Y. (2016). Sequencing of the litchi downy blight pathogen reveals it is a *Phytophthora* species with downy mildew-like characteristics. Mol. Plant Microbe Interact..

[34] Zhao X., Feng W., Zhu X., Li C., Ma X., Li X., Zhu X., Wei D. (2019). Conserved Autophagy Pathway Contributes to Stress Tolerance and Virulence and Differentially Controls Autophagic Flux Upon Nutrient Starvation in *Cryptococcus neoformans*. Front. Microbiol..

[35] Zhu X.M., Li L., Wu M., Liang S., Shi H.B., Liu X.H., Lin F.C. (2019). Current opinions on autophagy in pathogenicity of fungi. Virulence.

[36] Zhu X.M., Liang S., Shi H.B., Lu J.P., Dong B., Liao Q.S., Lin F.C., Liu X.H. (2018). VPS9 domain-containing proteins are essential for autophagy and endocytosis in *Pyricularia oryzae*. Environ. Microbiol..

