# Supplementary material for: Autophagy-Related Gene PlATG6a Is Involved in Mycelial Growth, Asexual Reproduction and Tolerance to Salt and Oxidative Stresses in Peronophythora litchii
Source: Int J Mol Sci. 2022 Feb 6;23(3):1839. doi: 10.3390/ijms23031839 (PMC8836449; doi:10.3390/ijms23031839)
Supplement: Supplementary file 1 [file ijms-23-01839-s001.zip › ijms-1476259-supplementary figures.pdf]

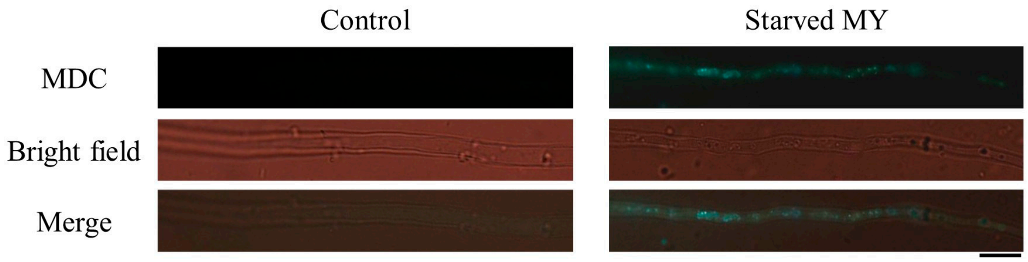

Figure S1: Visualization of autophagic activation with MDC in *P. litchii* mycelia (MY). Wide-type mycelia were incubated in CJA medium for 24 h. After 3 washes and incubation with sterile distilled water for 4 hours starvation, the mycelia were stained with MDC and analyzed by microscope. Bars=10 μm.

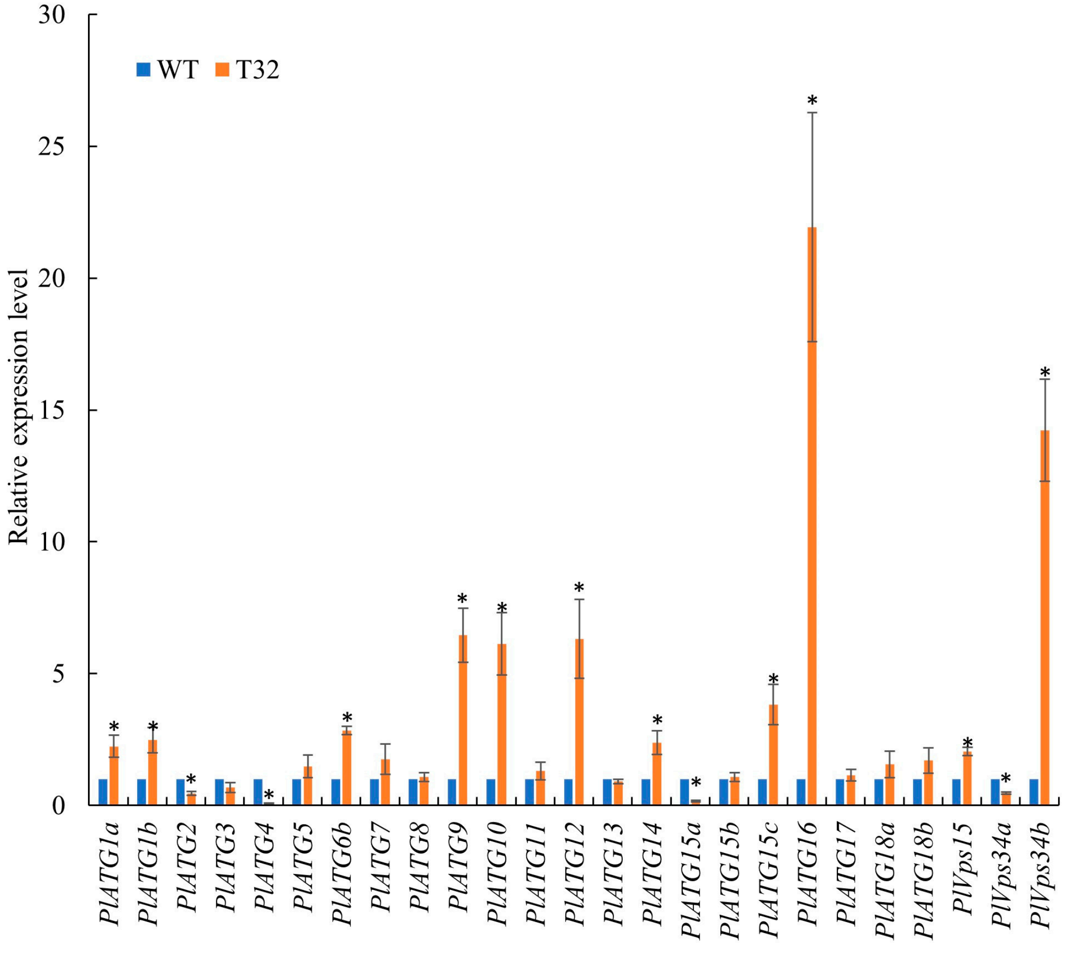

Figure S2: qRT-PCR measurement of relative transcript level of ATG genes in wild-type and *PlATG6a* mutant. Data are mean ± SD (n = 9). Asterisks represent significant differences vs WT (* $p < 0.05$) based on *t*-test.

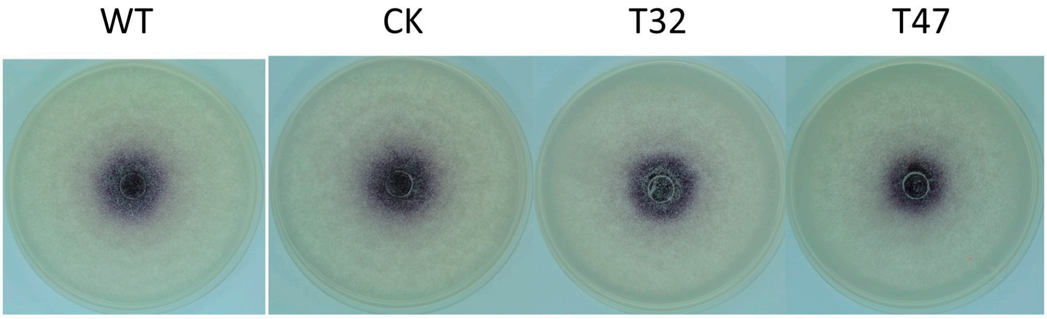

Figure S3: Laccase activity assay. Mycelial mats of WT, CK and Δ*platg6a* mutants were inoculated on lima bean agar (LBA) media containing 0.4 mM ABTS. Photographs were taken 7 days after inoculation.
